# How Cognitive Strengths Compensate Weaknesses Related to Specific Learning Difficulties in Fourth-Grade Children

**DOI:** 10.3389/fpsyg.2021.552458

**Published:** 2021-02-24

**Authors:** Marije D. E. Huijsmans, Tijs Kleemans, Evelyn H. Kroesbergen

**Affiliations:** Behavioural Science Institute, Radboud University, Nijmegen, Netherlands

**Keywords:** children, cognitive skills, comorbidity, compensation, learning difficulties, mathematics, reading, strengths and weaknesses

## Abstract

The goal of the present study was to investigate whether children’s cognitive strengths can compensate the accompanied weaknesses related to their specific learning difficulties. A Bayesian multigroup mediation SEM analysis in 281 fourth-grade children identified a cognitive compensatory mechanism in children with mathematical learning difficulties (*n* = 36): Children with weak number sense, but strong rapid naming performed slightly better on mathematics compared to peers with weak rapid naming. In contrast, a compensatory mechanism was not identified for children with a comorbid mathematical and reading difficulty (*n* = 16). One explanation for the latter finding could relate to the lack of ability to compensate, because of the difficulties these children experience in both academic domains. These findings lead to a new direction in research on learning difficulties in mathematics and/or reading by suggesting that children with a learning disability each have a unique profile of interrelated cognitive strengths and weaknesses. Children might compensate with these strengths for their weaknesses, which could lead to (small) learning gains in the affected domain.

## Introduction

Primary school children’s academic performance is characterized by great individual variation, and even within the group of children with a specific learning difficulty there is much heterogeneity (Moll et al., 2018). Children who experience learning difficulties, for example in mathematics or reading, each may have their own unique profile of cognitive weaknesses and strengths. Although previous research has to some extent recognized cognitive strengths in relation to learning difficulties (e.g., Toffalini et al., 2017), the main body of empirical research on learning difficulties solely investigated the weaknesses associated with them (for meta-analyses see Schwenk et al., 2017; Araújo and Faísca, 2019). Nonetheless, children may use cognitive strengths to compensate for their cognitive weaknesses, to prevent the development of more severe learning difficulties. The present study aimed to investigate children’s cognitive strengths as potential compensatory mechanisms for cognitive weaknesses related to their performance on mathematics and reading.

Research so far has made a significant contribution in identifying cognitive skills related to mathematics. Mathematics is defined as problem solving in the domains of proportions and geometry, including—but not limited to—calculations with fractions and measurements (Mullis et al., 2016). Variation in mathematics performance usually results from individual differences in number sense (Geary, 2011), working memory (Passolunghi and Siegel, 2004), and non-verbal reasoning (Kleemans et al., 2018). Number sense is defined as the capacity to recognize and understand symbolic numbers and non-symbolic numerosities (Dehaene et al., 1993, 2003), and has been found to play a key role in mathematics (Sasanguie et al., 2013). Working memory involves the temporal storage, processing and recollection (i.e., the executive function of updating) of verbal and visuospatial information (Passolunghi and Siegel, 2004; Alloway et al., 2009), and has been identified as a second cognitive factor in mathematics. However, reported effect sizes range from small (cf., geometry; Giofrè et al., 2014) to medium (cf., fractions; Hecht et al., 2003). Finally, non-verbal reasoning—or general intellectual ability—entails understanding of logical structure (Stock et al., 2009), and is strongly related to mathematics (with large effect sizes; Seethaler et al., 2011). Additionally, fact retrieval (i.e., automatizing and memorizing whole-number operations) is a prerequisite for advanced mathematics performance and acts as a mediator between effects of the cognitive skills on mathematics performance (Cirino et al., 2016).

Weaknesses in a cognitive skill related to mathematics often result in low mathematics performance. This idea corresponds with multiple-deficit models, wherein it is assumed that a specific learning difficulty develops as a result of a summation of its accompanying cognitive weaknesses (see e.g., Pennington, 2006; McGrath et al., 2011; Willcutt et al., 2013). Children with low math abilities thus would display weaknesses in number sense, working memory and/or non-verbal reasoning. However, some children experience (additional) weaknesses in phonological awareness and/or rapid naming, which originally are reading-related cognitive skills that also have become evident as predictors of mathematical difficulties (Vukovic and Lesaux, 2013). Other linguistic skills have been related to mathematics as well, such as grammatical ability (Kleemans et al., 2018), vocabulary (Purpura et al., 2017), decoding (De Smedt et al., 2010), and reading comprehension (Björn et al., 2016). These skills may either be directly associated with mathematics, or through their interaction with phonological awareness and rapid naming. When taking such cognitive variables into perspective, there now seem to be multiple alternative pathways to being (un)able to perform mathematics, which makes it difficult to predict mathematics performance from a unique set of (cognitive) skills (LeFevre et al., 2010). The averaged findings that result from such studies thus may not apply to all children within a group, as they all may have their own unique profile of cognitive weaknesses and strengths.

In a similar way, variation in reading performance (i.e., accurately decoding words and pseudo-words at an appropriate rate; Hasbrouck and Glaser, 2012) has consistently been linked to individual differences in phonological processing (i.e., phonological awareness and rapid naming; Landerl et al., 2009; Willcutt et al., 2013). Phonological awareness can be defined as the conscious process of recognizing and manipulating (i.e., deletion and segmentation of) sound segments, and is positively related to reading (Vellutino et al., 2004). Rapid naming refers to the capacity to quickly access and retrieve information from memory, and can be subdivided into alphanumeric (i.e., naming digits and letters) and non-alphanumeric (i.e., naming colors and pictures) skills (Willburger et al., 2008). Reaction times for (non-) alphanumeric rapid naming are negatively related to reading (Vellutino et al., 2004).

Children with comparable cognitive weaknesses can even vary in the severity of their learning difficulties (Huijsmans et al., under review). This clearly indicates that some children also have strengths in at least one other related cognitive skill, i.e., a *compensatory* mechanism to reduce the severity of their cognitive weaknesses. Compensation in the current study is defined as the ability to use an alternative (cognitive) skill to counteract a deficit in a closely related skill in order to maximize learning outcomes. This does not necessarily mean that a child with such compensatory strengths can fully overcome their learning problems, but we believe that the adverse effects of a cognitive deficit can be reduced by a cognitive strength.

Few empirical studies do explicitly report on cognitive strengths in children with learning difficulties, and those who did were limited to the assessment of reading disabilities only (e.g., Heim et al., 2008; Haft et al., 2016), or were restricted by only studying the intellectual profiles of various learning problems (Toffalini et al., 2017). Strengths in these studies, as well as in others (Ansari et al., 2003; Koriakin et al., 2017; Liu et al., 2017), have generally been defined as ‘relative strengths,’ meaning that these children display above average performance on a cognitive skill compared to other children with similar characteristics (e.g., a learning difficulty). Following this line of defining cognitive strengths, the same definition was used in the present study. Based on compelling evidence that phonological processing skills are related to mathematics (Berch and Mazzocco, 2007; Vukovic and Lesaux, 2013), it could be argued that strengths in phonological processing skills (i.e., phonological awareness and rapid naming) could act as a compensatory mechanism in mathematics performance. These cognitive skills might work on mathematics through related underlying cognitive deficits on for instance number sense and working memory. Children with such cognitive deficits may rely more on other cognitions when solving math problems. Their lack of understanding of number and numerosity (i.e., number sense) may to some extent be compensated by the ability to quickly retrieve procedural facts from long-term memory (i.e., rapid naming) to facilitate problem solving. Likewise, working memory and phonological awareness both enable children to manipulate (numerical) information (e.g., backwards recall or segmentation and blending, respectively), which can aid their math performance as well. The fact that children with specific mathematical difficulties mostly show weaknesses in number sense, working memory, and non-verbal reasoning (Slot et al., 2016), might indicate that a strength in phonological processing is a likely candidate for compensation of number sense or working memory weaknesses to prevent more severe math problems. In contrast, a strength in working memory could be a candidate for compensation of phonological deficits to reduce the severity of reading difficulties, because working memory has less consistently been related to reading than phonological awareness and rapid naming (Baddeley, 2003).

Given the fact that mathematics and reading show some overlap in terms of cognitive predictors (i.e., phonological awareness, rapid naming, and working memory; Wilson et al., 2015), it could be expected that a deficit in those cognitive skills might result in a comorbid mathematics and reading learning difficulty. Children with such a comorbid learning difficulty, on average, display the poorest academic outcomes in the domains of mathematics and reading compared to other children, despite intelligence being within the normal range (Landerl et al., 2009). For them, relying on compensatory cognitive skills when performing mathematics or reading tasks might not be possible, because cognitive strengths (relative to their peers) associated with mathematics and reading are less available to children with a comorbid learning difficulty (Jordan, 2007).

### The Present Study

Although cognitive strengths of children with specific learning difficulties have occasionally been recognized in recent studies, research often neglects to discuss the important implications of these strengths. This seems to be a misrepresentation of reality, because children’s cognitive strengths may in fact act as compensatory mechanisms against developing a comorbid learning deficit. Therefore, rather than just emphasizing children’s cognitive weaknesses as a marker of the development of learning deficits, the present study aimed to investigate children’s cognitive strengths as potential compensatory mechanisms for their cognitive weaknesses related to mathematics and/or reading proficiency.

It was hypothesized that children with either low mathematics performance, low reading performance, or a combination of both, show different compensatory mechanisms with respect to their learning difficulty. To examine this hypothesis, we assessed four different combinations of academic performance on mathematics and reading: Typical developing (TD) children, children with a specific learning difficulty in mathematics (MLD) or in reading (RLD) (i.e., below the 25th percentile on mathematics or reading respectively), and children with comorbid mathematical and reading learning difficulties (MRLD; i.e., below the 25th percentile on both mathematics and reading). Notice that we used a broad definition of learning difficulties, instead of just the inclusion of children with a diagnosis of dyscalculia or dyslexia. The reason for this approach is that it allowed us to investigate learning difficulties and associated cognitive strengths at the lower end of the continuum (Murphy et al., 2007). This interval includes the children wherein learning difficulties may be partly compensated, which may be a reason why they are not diagnosed with dyscalculia or dyslexia.

In each of these groups, we assessed which cognitive skills had the strongest effects on mathematics and reading. For TD-children it was expected that number sense, working memory, and non-verbal reasoning have the strongest effect on mathematics. Fact retrieval might mediate the effect between these cognitive skills and mathematics. We expected phonological awareness and rapid naming to have the strongest effects on reading. For children with a specific learning difficulty on mathematics and/or reading, it was expected that different cognitive skills would show a stronger effect on the academic performance of interest (e.g., mathematics in the MLD group) compared to TD-children, because there is little variability on the regular predictors. Therefore, we investigated whether other medium to strong cognitive effects could be identified as a cognitive strength to compensate for cognitive weaknesses in the learning difficulty groups. Phonological processing skills might act as a compensatory mechanism for mathematics in children with low math abilities, because some children might show relatively strong performance on those cognitive skills in spite of their learning difficulty. This will result in better performance on mathematics compared to their peers without such a compensatory cognitive strength. Compensatory effects of number sense, working memory, and non-verbal reasoning are unlikely, as children with math problems often experience difficulties with these cognitive skills, and thus will show little variation (i.e., smaller effects) for those variables. In contrast, working memory might have the strongest effect on reading as a compensatory mechanism for children with low reading abilities, because for some of them their working memory performance might be relatively strong compared to peers. Children with reading problems are likely to show the least variance (and thus smaller effects) on phonological awareness and rapid naming. As number sense and non-verbal reasoning play a minor role in reading, strength in working memory is the most likely candidate for compensation within reading. Finally, compensatory effects might be non-existent for children with comorbid learning difficulties, because they have low performance on all cognitive skills (i.e., little variance and thus smaller effects), and therefore cannot rely on cognitive strengths.

## Materials and Methods

### Participants and Procedure

The present study reported on data collected during the first measurement of an ongoing longitudinal study on the predictors of numerical development. The final sample included 281 fourth-grade children (*M*_age_ = 9.3 years, *SD* = 0.5) from eleven Dutch primary schools. The study was approved by the institutional ethics review board, and parental active informed consent was obtained prior to data collection. Exclusion criteria included any physical, behavioral or learning disability other than MLD or RLD, as reported by the teacher. All participants spoke Dutch fluently. Missing data for 61 children were handled using multiple imputation (Rubin, 1987). Missing values were estimated ten times, and pooled into one aggregated score. Independent and dependent variables were imputed separately.

Four groups were created for further analyses, using the Dutch national standardized tests for mathematics (CITO Rekenen-Wiskunde, CITO-RW, *Mathematics test*; Janssen et al., 2010) and reading (Cito Drie Minuten Test, DMT, *Three Minutes Test*; Verhoeven, 1992). Children with a mathematical learning difficulty (MLD; *n* = 36) scored at or below the 25th percentile on the CITO-RW and above the 25th percentile on the CITO-DMT. Children with a reading learning difficulty (RLD; *n* = 42) scored at or below the 25th percentile on the CITO-DMT and above the 25th percentile on the CITO-RW. Children with a mathematics and reading learning difficulty (MRLD; *n* = 16) scored at or below the 25th percentile on both the CITO-RW and the CITO-DMT. Finally, typically developing children (TD; *n* = 168) scored above the 25th percentile on both tests. Parents of nineteen children (7%) did not permit the school to share their children’s CITO-scores. Therefore, these children were excluded from further analysis.

Background characteristics for the children in the TD, MLD, RLD, and MRLD groups are shown in Table 1. There were no age differences between groups (*BF*s < 2.31; anecdotal support; Jeffreys, 1961). Gender was equally distributed across groups, with the exception that there were more girls than boys within the group of MLD-children (i.e., 72.2% girls). Most children in each group were Dutch, and the parents of one quarter to one third of the children per group were relatively highly educated (i.e., applied university or university). Ethnic background and SES did not differ across groups (χ^2^s < 12.93, *BF*s < 1).

**TABLE 1:** Background characteristics for the TD, MLD, RLD, and MRLD groups.

	TD (*n* = 168)	MLD (*n* = 36)	RLD (*n* = 42)	MRLD (*n* = 16)
Age (in months)	115.82 (5.25)	118.92 (6.52)	118.33 (5.63)	119.06 (4.94)
Gender (% girls)	45.2%	72.2%	50.0%	56.3%
Ethnicity (% Dutch)	93.6%	86.2%	91.9%	78.6%
SES (% higher-education)	33.3%	19.4%	35.8%	25.0%

*A Bayesian one-way ANOVA for age and Bayesian Chi-square tests for ethnicity and SES revealed that there were no initial group differences (*BF*s < 3). However, there were more girls in the MLD-group, compared to the TD-, RLD-, and MRLD-groups.*

The test battery lasted 3.5 h per child (spread across several school days), consisting of classroom measures (mathematics, fact retrieval, phonological awareness, and non-verbal reasoning), computerized measures (number sense and working memory), and individual measures (decoding and rapid naming). All measures were administered by trained students who followed a standardized protocol. Classroom measures were administered in three test blocks of 45 min each (i.e., 2 h and 15 min in total), counterbalanced across schools. Block A and B consisted of Parts 1 and 2 of the mathematics task, respectively. In Block C, the tasks for fact retrieval, phonological awareness, and non-verbal reasoning were administered consecutively. Computerized measures were self-reliant: Tasks were administered in approximately 45 min (15 min for number sense, and 30 min for working memory) in a group-wise setting of approximately six children per subgroup. Individual measures were administered within 20 min per child in a quiet room, and included tests for decoding and rapid naming.

### Measures

#### Academic Variables

##### Mathematics (for classification)

The CITO-RW (Janssen et al., 2010) was used for classification of children as MLD or MRLD. This task is a Dutch national standardized test for mathematics with different, grade-appropriate versions (50–54 items per version) that are administered twice a year by the classroom teacher. The scores obtained in the middle of fourth-grade were used in the present study. Internal consistency was good (*a* > 0.91; Evers et al., 2009–2012).

##### Mathematics (for analyses)

An adapted version of the Schoolvaardigheidstoets Rekenen-Wiskunde (SVT-RW, *School Achievement Test for Mathematics*; De Vos and Milikowski, 2012) was used to assess advanced mathematics. Items from the original SVT-RW for grades 4, 5, and 6 were selected (i.e., to prevent ceiling effects) and were combined into one task (e.g., 3 km + 300 m = ___ m; calculate the surface of ‘this’ object). Additional items from an older, no longer used version of the Dutch national test for mathematics (CITO-RW; Janssen et al., 2005), and the *Fraction Competency Test* (FCT; Brown and Quinn, 2007) were added to obtain a comprehensive assessment of children’s mathematical skills. An exemplary item for the CITO-RW was ‘mom buys four tickets of 15 euros each, and pays with 100 euros. How much change does she receive?’, and for the FCT 3 – 1/5 = __. The final mathematics paper-and-pencil task was administered in the classroom. The task consisted of two parts with 61 open-ended computational problems in total, and a time limit of 45 min per part. The computational problems contained little text to prevent that children should rely on their reading skills. We ran several analyses to assess the mathematics task at the item level. Combined findings regarding (1) internal validity using item response theory (two-parameter Birnbaum model) in the open-source R software (version 3.4.4), and (2) fit to the latent factor by means of factor analysis in SPSS (version 23.0), resulted in the removal of five items that either were too difficult, discriminated poorly, and/or did not fit to the latent factor. Each correct answer yielded one point, summing to a total maximum score of (61 – 5 =) 56 points. Internal consistency in the present study was good (α = 0.89).

##### Reading (for classification)

The CITO-DMT (Verhoeven, 1992) was used for classification of children as RLD or MRLD. This task is a Dutch national standardized test for word reading with different, grade-appropriate versions (three reading cards per version) that are administered twice a year by the teacher. Each version consists of three reading cards with 150 words per card. Words increase in complexity across cards, shifting from monosyllabic words on the first card to polysyllabic words on the third card. The scores obtained in middle fourth-grade were used in the present study. Internal consistency was good (*a* = 0.80; Evers et al., 2009–2012).

##### Reading (for analyses)

Children’s reading was assessed individually using two measures. Word decoding was measured with the Eén Minuut Test *(EMT, One Minute Test*; Brus and Voeten, 1999), and pseudoword decoding was measured with the Klepel (Van den Bos et al., 1994). In both tasks, children had to accurately read as many unrelated (pseudo-)words as they could within 1 min. To increase difficulty level, word length increased from one to four syllables. The number of correctly read (pseudo-)words for each task was the raw score, with a maximum of 116 words per task. Scores from both tasks were averaged into one score for decoding. Internal consistency was good, with α = 0.90 for the EMT and α = 0.92 for the Klepel (Evers et al., 2009–2012).

##### Fact retrieval

The Tempo Test Automatiseren (TTA, *Speeded Arithmetic Test*; De Vos, 2010) was used in the participants’ classroom to assess children’s fact retrieval. The four subtests addition, subtraction, multiplication and division each included 50 paper-and pencil problems of increasing complexity. Children were instructed to solve as many problems per subtest as possible within 2 min. Each correct answer yielded one point, summing to a maximum score of 200 points. Internal consistency for all subtests was at least sufficient (α’s > 0.78; Evers et al., 2009–2012).

#### Cognitive Variables

##### Number sense

Number sense was assessed with the computerized *Dutch Assessment battery for Number Sense* (DANS; Friso-Van den Bos et al., 2015). There were two subtests: Symbolic comparison and non-symbolic comparison. Stimuli were presented at random using E-prime software (Version 2.0). The symbolic and non-symbolic comparison tasks required participants to rapidly indicate which of two numbers (symbolic) or sets of dots (non-symbolic) was the largest using key-press. Average size and range for symbolic number were *M* = 49.17, Range = 10; 96, and for non-symbolic numerosity *M* = 52.02, Range = 14; 97. The mean and range of the ratios were *M* = 0.75, Range = 0.63; 0.88, and *M* = 0.78, Range = 0.63; 1.00 for symbolic number and non-symbolic numerosity, respectively. Dot size, area, and density were manipulated in the non-symbolic condition using the approach of Dehaene et al. (2005), to ensure that the responses are being associated with quantity instead of dot patterns. After a training block, testing blocks with 33 and 43 items, respectively, of varying difficulty were administered in random order. Average reaction time in ms for the correct trials was used for further analysis, because accuracy scores produced ceiling effects in the symbolic condition (*M* = 32.43, *SD* = 2.52; non-symbolic condition, *M* = 27.92, *SD* = 3.50). Internal consistency of the comparison tasks is good (α’s > 0.84; Kline, 1999).

##### Working memory

The online computerized tasks *Lion game* and *Monkey game* were used to assess visuospatial and verbal working memory, respectively. In the Lion game, children had to remember the locations of pictures of colored lions within a 4 × 4 matrix (Van de Weijer-Bergsma et al., 2015a). Children were presented with 20 items (five levels of four items) and for each item had to indicate the last location(s) of one or more lion(s) of a specific color (e.g., red, blue, yellow, green, or purple). In the monkey game, children had to remember and recall spoken familiar words in reversed order (Van de Weijer-Bergsma et al., 2016). Children were presented with 20 items (five levels of four items), and by mouse click on written words in a 3 × 3 matrix were able to indicate the correct backwards order of the spoken words. For both tasks, the average proportion of correctly recalled items was used as raw score. Internal consistency for both tasks was good (α’s > 0.87; Van de Weijer-Bergsma et al., 2015b, 2016).

##### Phonological awareness

A phonological awareness task (Knoop-van Campen et al., 2018) was administered in the classroom. In the 18-item deletion subtask, children had 4 s to delete a letter (e.g., ‘s’) from a spoken word (e.g., ‘small’), and cross the corresponding picture (e.g., ‘mall’, with distracters ‘ball’ and ‘wall’). In the 12-item spoonerism subtask, five pictures were shown and children had 5 s to switch the first letters of two verbally presented words (e.g., ‘mouse’ and ‘heat’ become ‘house’ and ‘meat’) by crossing the corresponding pictures. On each task, one point was given per correct answer [cf. a maximum score of (2 ^∗^ 12 =) 24 points]. Internal consistency was sufficient (α = 0.70, Knoop-van Campen et al., 2018).

##### Rapid naming

The Continu Benoemen subtest of the Continu Benoemen en Woorden Lezen test *(CB&WL, Continuous Naming and Word Reading*; Van den Bos and Lutje Spelberg, 2007) was administered individually to measure rapid naming. It exists of four subtests with five high frequent items: Colors (black, yellow, red, green, blue), digits (2, 4, 5, 8, 9), pictures (tree, chair, duck, scissors, bike), and letters (d, o, a, s, p). Children were instructed to rapidly and accurately name these visually presented items. All items were at random presented 10 times (i.e., 50 items per subtest, 200 items in total). Averaged overall naming time in seconds was used as raw score. Split-half reliability and test–retest reliability were sufficient (α’s > 0.75; Evers et al., 2009–2012).

##### Non-verbal reasoning

Raven’s Standard Progressive Matrices were used to assess non-verbal reasoning (Raven, 1976). This task consists of 60 visual patterns (i.e., five sets of 12 items), with increasing difficulty. In the first set, one part was missing for each item. Children were asked to select the missing part to logically complete the design out of six alternatives. In the remaining sets, four to nine pattered figures were presented, from which the final figure was missing. Children selected the missing figure out of six to eight alternatives. The number of correct answers were counted, summing to a maximum score of 60 points. Internal consistency was good (α > 0.90; Raven et al., 1998).

### Analysis Strategy

#### Preliminary Analyses

All variables were approximately normally distributed (standardized | skewness| and | kurtosis| < 3.0). This was computed by dividing the skewness and kurtosis statistics (obtained in SPSS, version 25) by their standard errors. Outliers that diverged more than three standard deviations from the mean (>| 3.29|) were winsorized. Subvariables of all cognitive constructs were correlated in the total sample (*BF*s > 16.07; strong support; Jeffreys, 1961). This was the case for number sense (*r* = 0.34 for non-symbolic and symbolic comparison), working memory (*r* = 0.16 for verbal and visuospatial working memory), phonological awareness (*r* = 0.35 for deletion and spoonerism), and rapid naming (*r* = 0.64 for alphanumeric and non-alphanumeric rapid naming). It should be noted, however, that the correlation between both working memory constructs was relatively weak, but both observed variables were still combined into one latent variable in further analyses in line with previous research (LeFevre et al., 2013; Giofrè et al., 2014; Gray et al., 2017). There was strong support (*BF*s > 7313174) for correlations between working memory, phonological awareness, and non-verbal reasoning, see Table 2. The other cognitive skills were not related to each other, thus covariances for those associations were set to zero in further analyses.

**TABLE 2:** Correlations between the cognitive skills (*n* = 262).

	Number	Working	Phonological	Rapid	Nonverbal
	sense	memory	awareness	naming	reasoning
Number sense^a^					
Working memory	–0.06				
Phonological awareness	–0.11	0.37*			
Rapid naming^a^	0.13	–0.01	–0.14		
Nonverbal reasoning	0.08	0.39*	0.36*	0.05	

*BF > 10 (strong support); see Jeffreys, 1961. ^*a*^Reaction time measures.

#### Statistical Analyses

Bayesian structural equation modeling (BSEM) was conducted to examine cognitive compensatory mechanisms in mathematics and decoding, using the blavaan-package (Merkle and Rosseel, 2018) in open-source R software (version 3.6.1). A Bayesian approach was chosen because this allowed us to estimate a complex multigroup mediation SEM model within a small sample: There are few children with a (specific) learning difficulty within a regular sample of primary school children (as explained in the introduction). Another advantage of the Bayesian technique is that we could specify informative priors. See Van de Schoot and Depaoli (2014) and Van de Schoot et al. (2014) for a further (introductory) discussion of the advantages of Bayesian analyses. Unique effects between the cognitive skills and mathematics, and between the cognitive skills and decoding have already been established in previous empirical research and including this information as priors in our comprehensive model lead to more reliable results. Beta’s and precision scores (corrected for sample size) were obtained from the data reported in those studies, and were used to specify the limits to the normal distribution of the priors, see Appendix. Prior information was retrieved from mixed samples (e.g., TD and MLD) as much as possible, because the same values were used in all models as we employed a multigroup approach. BSEM does not require the same assumptions as frequentist SEM (e.g., asymptotic normality), because exact posterior distributions can be estimated (instead of assumed) for any functional of the parameters and latent variables (Levy, 2011).

First, a Bayesian confirmatory factor analysis (BCFA; measurement model) was conducted on the whole sample to depict indicators of the standardized latent exogenous cognitive skills (i.e., number sense, working memory, phonological awareness, and rapid naming), and the standardized latent endogenous behavioral skills (i.e., mathematics, decoding, and fact retrieval). Non-verbal reasoning had a single indicator and was therefore set to ‘1’. Number sense and rapid naming were reaction time measures, and were recoded prior to the analyses. Second, a Bayesian mediation path analysis (BSEM; structural model) was carried out to display the predictors of mathematics and decoding, once within the full sample (reference model), and once within TD, MLD, RLD, and MRLD children (multigroup model). Fact retrieval was included in the model as mediator between the cognitive skills and mathematics. Goodness of fit of the models was examined using the posterior predictive *p*-value (ppp ≥ 0.05 indicates good fit; Meng, 1994), and models were compared using several information criteria (dic, waic, and looic; smaller values indicate better fit of the model to the data compared to a model with larger values; Spiegelhalter et al., 2002). All Bayes Factors (i.e., the test statistic) were interpreted according to the guidelines by Jeffreys (1961), see Table 3.

**TABLE 3:** Interpretation of the Bayes factor (Jeffreys, 1961).

Bayes factor			Interpretation
	>	100	Decisive evidence for H_1_
30	–	100	Very strong evidence for H_1_
10	–	30	Strong evidence for H_1_
3	–	10	Substantial evidence for H_1_
1	–	3	Anecdotal evidence for H_1_
	1		No evidence
1/3	–	1	Anecdotal evidence for H_0_
1/10	–	1/3	Substantial evidence for H_0_
1/30	–	1/10	Strong evidence for H_0_
1/100	–	1/30	Very strong evidence for H_0_
	<	1/100	Decisive evidence for H_0_

To explore compensatory mechanisms, Bayesian independent samples t-tests were conducted in R using the BayesFactor-package (Morey et al., 2018). This exploratory analysis was carried out to examine whether children with a cognitive strength—as opposed to a weakness in that same cognitive skill—can compensate for a related cognitive weakness associated with their learning difficulty. In line with Ansari et al. (2003), Heim et al. (2008), Haft et al. (2016), Koriakin et al. (2017), Liu et al. (2017), and Toffalini et al. (2017), a strength was defined as +1 SD relative to the sample mean, and a weakness as –1 SD relative to the sample mean.

## Results

### Descriptive Statistics

Descriptive statistics for all behavioral and cognitive measures are displayed in Table 4. Interesting to note is that mathematics performance in the MLD- and MRLD-group was significantly lower than the RLD-group, which in turn was weaker compared to TD-group. In contrast, performance on decoding of the RLD- and MRLD-group was significantly weaker than for the TD- and MLD-group. Fact retrieval of the MLD- and MRLD-group was significantly lower than for the TD-group. However, fact retrieval skills of the RLD-group were similar to those of the MLD- and MRLD-group.

**TABLE 4:** Means (standard deviations) for the TD, MLD, RLD, and MRLD groups.

	TD (*n* = 168)		MLD (*n* = 36)		RLD (*n* = 42)		MRLD (*n* = 16)	
	*M (SD)*	*Min; Max*	*M (SD)*	*Min; Max*	*M (SD)*	*Min; Max*	*M (SD)*	*Min; Max*
**Mathematics**	20.62 (7.36)^a^	6; 41	9.34 (5.08)^c^	2; 28	17.29 (7.49)^b^	5; 34	9.63 (5.02)^c^	3; 23
**Decoding**	53.04 (9.39)^a^	32.5; 82	51.79 (9.56)^a^	27.5; 72	36.69 (8.00)^b^	24.5; 60	35.09 (8.20)^b^	23; 59
**Fact retrieval**	126.34 (30.47)^a^	53; 196	91.38 (26.70)^b^	44; 174	106.51 (30.27)^b^	49; 176	84.63 (33.87)^b^	31; 156
**Number sense (ms)**								
Symbolic	1167.73 (259.64)^a^	600.00; 2000.00	1195.47 (224.02)^a^	851.24; 1774.18	1171.74 (192.13)^a^	829.32; 1624.64	1474.71 (255.02)^b^	847.03; 2000.00
Non-symbolic	1291.90 (300.28)^a^	505.30; 2000.00	1042.07 (308.28)^b^	500.00; 1683.57	1322.17 (283.27)^a^	668.05; 2000.00	1323.19 (317.37)^a^	888.98; 1728.29
**Working memory (*p*)**								
Verbal	0.58 (0.09)^a^	0.30; 0.85	0.48 (0.15)^b^	0.20; 0.80	0.51 (0.13)^b^	0.20; 0.74	0.48 (0.08)^b^	0.36; 0.62
Visuospatial	0.74 (0.12)^a^	0.31; 0.99	0.69 (0.14)^a^	0.30; 0.93	0.74 (0.12)^a^	0.44; 0.96	0.68 (0.15)^a^	0.35; 0.95
**Phonological awareness**								
Deletion	15.55 (2.51)^a^	7; 18	14.12 (3.05)^b^	7; 18	14.62 (2.30)^ab^	9; 18	13.38 (2.13)^b^	9; 17
Spoonerism	16.09 (4.21)^a^	3; 24	13.12 (3.95)^b^	3; 20	13.32 (4.55)^b^	6; 22	12.38 (4.47)^b^	6; 21
**Rapid naming (sec)**								
Alphanumeric	26.29 (4.45)^a^	18.15; 38.27	25.31 (3.81)^a^	15.79; 31.57	31.28 (6.14)^b^	21.36; 44.63	31.10 (7.06)^b^	20.45; 44.99
Non-alphanumeric	45.98 (6.62)^a^	32.27; 65.90	45.96 (6.58)^a^	34.10; 61.00	50.35 (7.67)^b^	34.54; 72.48	53.63 (9.57)^b^	40.20; 72.53
**Nonverbal reasoning**	41.11 (6.56)^a^	20; 54	33.41 (6.31)^b^	20; 44	39.85 (5.85)^a^	28; 51	33.53 (5.88)^b^	23; 42

*Note that group means with different superscripts are different (BF > 3). Bayesian ANOVA was employed to assess group differences.*

With respect to the mathematics predictors, for number sense the MRLD-group performed the worst (i.e., they had the slowest reaction times) on symbolic number sense. Contrary to our expectations, the MLD-group performed the best (i.e., quickest reaction times on correct trials) of all groups on non-symbolic number sense, which will be elucidated in the discussion section. Verbal working memory performance was significantly weaker in all learning-difficulty groups compared to TD-children, but visuospatial working memory did not differ across groups. Finally, non-verbal reasoning was significantly weaker in the MLD- and MRLD-group than in the TD- and RLD-group. Regarding the linguistic predictors, we found that phonological awareness was significantly weaker in the learning-difficulty groups compared to TD-children. Finally, rapid naming was significantly weaker in the RLD- and MRLD-groups compared in the TD- and MLD-groups. Variance across groups on all cognitive measures was quite similar.

#### Correlations

Correlations between the behavioral and cognitive skills of the overall sample are presented in Tables 5A, B. Mathematics had a significant positive correlation with working memory, phonological awareness, and non-verbal reasoning. Furthermore, mathematics was correlated to fact retrieval (*r* = 0.52, *BF* = 7.34^20^; strong support; Jeffreys, 1961). Fact retrieval itself had a significant negative correlation with number sense and rapid naming (i.e., slower reaction times indicate lower fact retrieval scores), and a significant positive correlation with working memory, phonological awareness and non-verbal reasoning. Finally, decoding had a significant negative correlation with rapid naming (i.e., slower reaction times indicate lower decoding scores), and a significant positive correlation with phonological awareness.

**TABLE 5A:** Correlations between latent behavioral skills and latent cognitive skills (*n* = 262).

	Number	Working	Phonological	Rapid	Nonverbal
	sense^a^	memory	awareness	naming^a^	reasoning
Mathematics	–0.12	0.39***	0.38***	–0.13*	0.51***
Decoding	–0.12	0.07	0.33***	–0.56***	0.00
Fact retrieval	–0.19*	0.22**	0.31***	–0.29***	0.21**

*BF = 1–3 (anecdotal support); **BF = 3–10 (substantial support); ***BF > 10 (strong support); see Jeffreys, 1961. ^*a*^Reaction time measures.

**TABLE 5B:** Correlations between observed behavioral skills and observed cognitive skills (*n* = 262).

	1	2	3	4	5	6	7	8	9	10	11	12	13	14	15	16
(1) Proportions																
(2) Geometry	0.273															
(3) Word decoding	0.097	0.148														
(4) Pseudoword decoding	0.063	0.101	0.850													
(5) Addition	0.201	0.395	0.403	0.448												
(6) Subtraction	0.181	0.496	0.366	0.374	0.839											
(7) Multiplication	0.130	0.311	0.411	0.394	0.678	0.703										
(8) Division	0.162	0.410	0.324	0.270	0.595	0.648	0.812									
(9) Symbolic comparison	–0.151	–0.276	–0.119	–0.103	–0.370	–0.392	–0.252	–0.259								
(10) Non-symbolic comparison	0.002	0.107	–0.040	–0.079	–0.004	0.043	0.047	0.058	0.348							
(11) Visuospatial WM	0.132	0.197	–0.002	–0.106	–0.015	–0.001	0.007	0.082	–0.055	0.000						
(12) Verbal WM	0.193	0.327	0.199	0.149	0.289	0.264	0.227	0.239	–0.280	0.125	0.200					
(13) Deletion	0.203	0.287	0.240	0.210	0.165	0.226	0.168	0.215	–0.197	0.022	0.190	0.322				
(14) Segmentation	0.125	0.277	0.290	0.291	0.212	0.244	0.222	0.227	–0.069	–0.081	0.207	0.312	0.361			
(15) Alphanumeric RAN	–0.040	–0.074	–0.605	–0.611	–0.426	–0.312	–0.321	–0.192	0.158	0.070	0.134	–0.050	–0.101	–0.084		
(16) Non-alphanumeric RAN	–0.066	–0.145	–0.451	–0.394	–0.344	–0.259	–0.259	–0.201	0.158	0.029	0.031	–0.133	–0.089	–0.150	0.655	
(17) Non-verbal reasoning	0.175	0.458	0.059	–0.025	0.145	0.213	0.131	0.222	–0.082	0.190	0.307	0.327	0.333	0.293	0.150	–0.019

### Bayesian Multigroup Mediation Structural Equation Model

**FIGURE 1:**
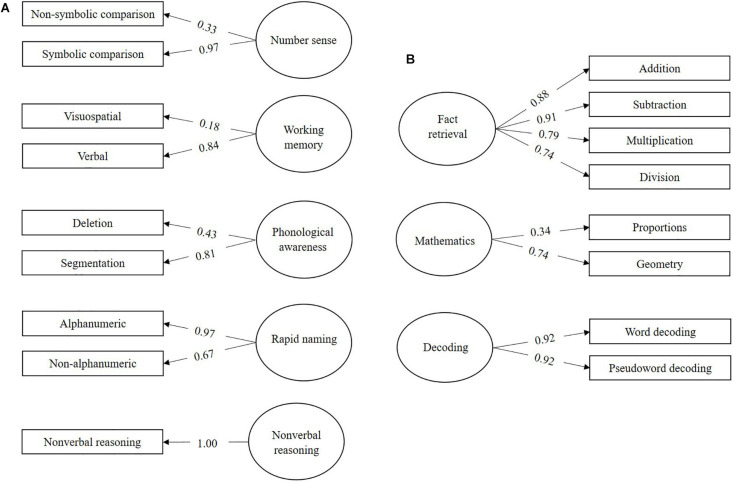
Measurement model (CFA) for **(A)** the cognitive skills and **(B)** the behavioral outcomes in the combined sample of all children. Denoted values are beta weights.

The combined sample of all children was used to create a measurement model (Figure 1) with subvariables of cognitive predictors (number sense, working memory, phonological awareness, rapid naming, and non-verbal reasoning), behavioral outcomes (mathematics and decoding), and behavioral mediator (fact retrieval). Model fit to the data was considered sufficient, ppp = 0.06.

Next, structural relations by means of informative priors were included in the measurement model to facilitate SEM. A Bayesian mediation SEM analyses was conducted on the combined sample of all children to create a reference model. A Bayesian multigroup mediation SEM analyses was conducted on the TD-, MLD-, RLD-, and MRLD-group, again using the measurement model that was retrieved from the combined sample of all children. The group factor was constructed such that the LD-groups were compared to the TD-group. Fit statistics and information criteria from the multigroup model were compared to the reference model, see Table 6, and revealed that the multigroup model was preferred because of better model fit and smaller values for the information criteria. In fact, the reference model showed poor fit to the data, and was therefore neither plotted nor interpreted in the present study.

**TABLE 6:** Information criteria for the comparison of the multigroup model to the reference model.

Model	ppp	DIC	WAIC	LOOIC
Multigroup	0.26	9963.78	10070.79	10079.98
Reference	0.00	10916.68	10933.02	10933.21
Sensitivity	0.48	9855.93	10018.36	10026.07

*Smallest values for information criteria indicate preferred model.*

**FIGURE 2:**
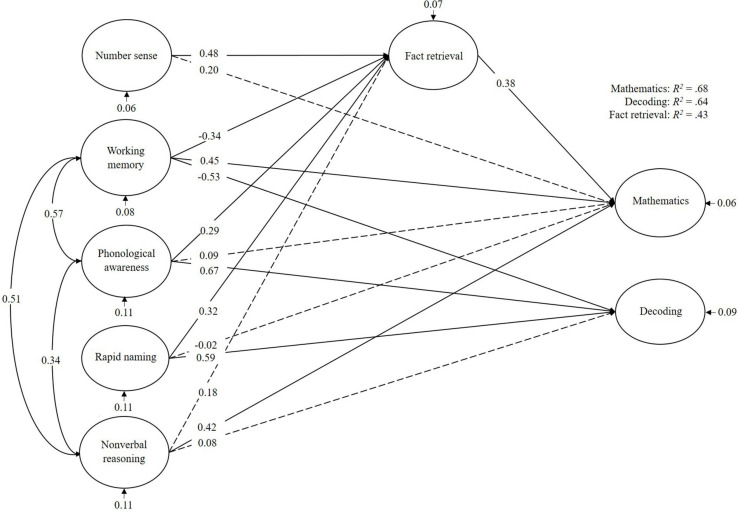
Bayesian structural equation model for typical developing children. Denoted values are beta weights.

For the TD-group (Figure 2), mathematics was mainly predicted by working memory and non-verbal reasoning. Fact retrieval did not mediate this effect, but number sense, working memory, phonological awareness, and rapid naming were predictors of fact retrieval. Decoding was mainly predicted by working memory, phonological awareness, and rapid naming. In total, the cognitive predictors explained 68% of the variance in mathematics, 64% of the variance in decoding, and 43% of the variance in fact retrieval.

**FIGURE 3:**
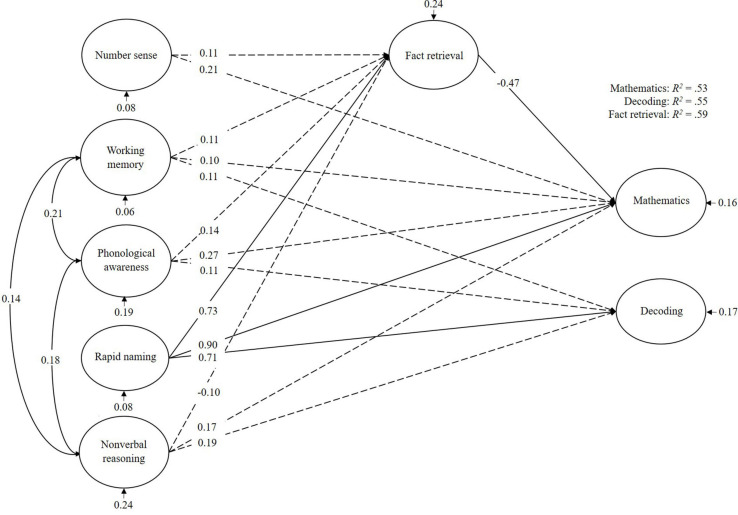
Bayesian structural equation model for children with weak math abilities (MLD). Denoted values are beta weights.

For the children with low math abilities (Figure 3), mathematics was mainly predicted by rapid naming. Fact retrieval mediated this effect. Decoding too was mainly predicted by rapid naming. In total, the cognitive predictors explained 53% of the variance in mathematics, 55% of the variance in decoding, and 59% of the variance in fact retrieval.

**FIGURE 4:**
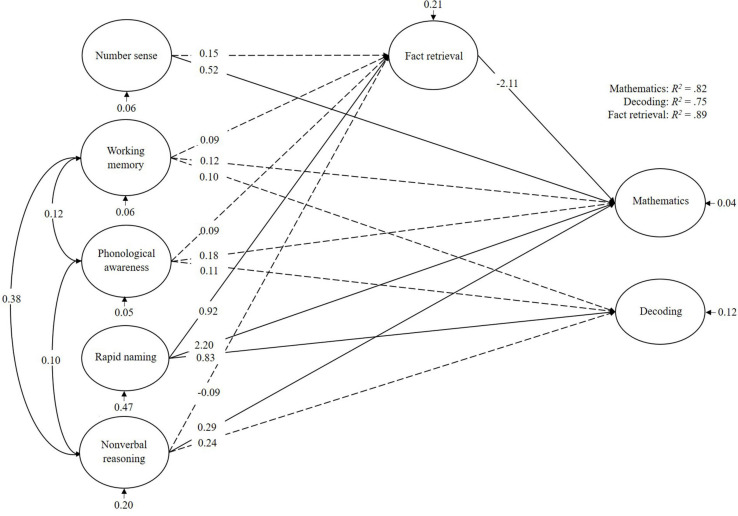
Bayesian structural equation model for children with weak decoding abilities (RLD). Denoted values are beta weights.

For the children with low decoding abilities (Figure 4), mathematics was mainly predicted by number sense, rapid naming, and non-verbal reasoning. Fact retrieval mediated this effect for rapid naming and number sense. Decoding too was mainly predicted by rapid naming. In total, the cognitive predictors explained 82% of the variance in mathematics, 75% of the variance in decoding, and 89% of the variance in fact retrieval.

**FIGURE 5:**
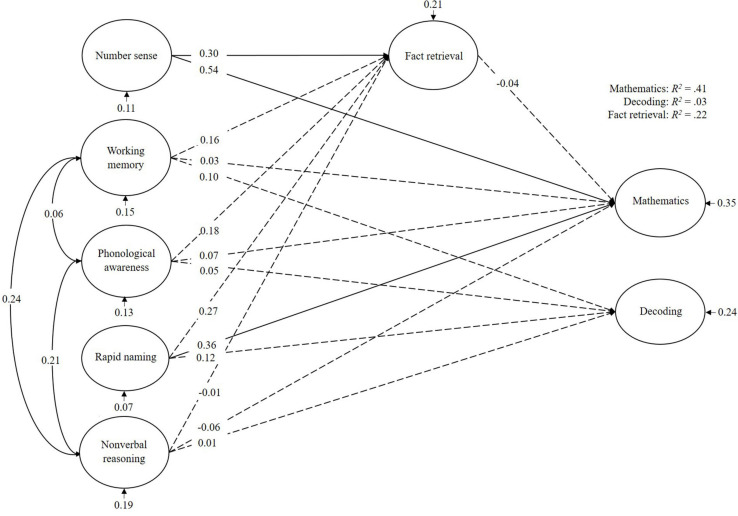
Bayesian structural equation model for children with comorbid learning difficulties (MRLD). Denoted values are beta weights.

For the children with mathematics and reading learning difficulties (Figure 5), mathematics was mainly predicted by number sense and rapid naming. Fact retrieval did not mediate this effect, but was predicted by number sense. Decoding was not predicted by any of the cognitive variables included in this model. In total, the cognitive predictors explained 41% of the variance in mathematics, 3% of the variance in decoding, and 22% of the variance in fact retrieval.

Indirect and total effects for mediation by fact retrieval are presented in Table 7. Within TD-children, direct effects of the cognitive skills on mathematics (especially for working memory and non-verbal reasoning) were stronger than indirect effects via fact retrieval. For the MLD-group and RLD-group, the effects of rapid naming, and rapid naming and number sense, respectively, on mathematics were mediated by fact retrieval, but the direction of these effects was negative and should therefore be interpreted with care: Children with higher scores on fact retrieval appeared to perform weaker on mathematics.

**TABLE 7:** Indirect and total effects (marked bold) for fact retrieval as mediator in the effects of the cognitive skills on mathematics.

	TD		MLD		RLD		MRLD	
	Indirect	Total	Indirect	Total	Indirect	Total	Indirect	Total
Number sense	0.18	**0.38**	–0.05	0.16	**–0.32**	0.20	–0.01	**0.53**
Working memory	–0.13	**0.32**	–0.05	0.05	–0.20	–0.08	–0.01	0.02
Phonological awareness	0.11	0.19	–0.07	0.20	–0.18	–0.01	–0.01	0.06
Rapid naming	0.12	0.10	**–0.35**	**0.55**	**–1.93**	0.26	–0.01	**0.35**
Non-verbal reasoning	0.07	**0.49**	0.05	0.21	0.18	**0.47**	0.00	–0.06

Lastly, to ensure that the priors did not affect our data substantially, a sensitivity analysis with non-informative priors was modeled. All other parameters were kept the same as in the main analysis. Fit statistics and information criteria are displayed in Table 6. Overall, results from the sensitivity analysis were quite similar to the main analysis. However, based on the sensitivity model it appears as if the priors have had some impact on the data. First, some effects were more extreme in the sensitivity analysis, whereas others were more tempered. To elaborate, the effects of working memory on mathematics and decoding were larger in the TD-group in the sensitivity model than in the main model, although they remained to be in the same direction. In contrast, the effects of number sense and rapid naming on mathematics were smaller in all four groups in the sensitivity model as opposed to the main model, but again the direction of the effects remained the same. Despite these shifts in the sizes of the effects, conclusions regarding those variables were the same for both the sensitivity analysis and the main analysis. In contrast, conclusions regarding the mediation effects were different based on how informative the priors were. Under the non-informative priors (i.e., sensitivity model), the effect of fact retrieval on mathematics became close to zero (but was still negative) in the MLD-group, and even switched directions in the RLD-group (i.e., became positive instead of negative) as opposed to the informative priors (i.e., main model). This finding will be further reflected on in the section “Discussion.” Overall, except for the mediation effects, data were not substantially affected by the priors.

Taken together, the Bayesian SEM model for the TD-children was matching findings from empirical research: Mathematics and fact retrieval were predicted by the mathematic cognitive skills, and also to some extent by the linguistic predictors. Decoding was predicted by these linguistic cognitive skills as well. For the children with MLD, rapid naming was the strongest predictor for mathematics, fact retrieval and decoding. Their rapid naming scores were at the same level as TD-children’s performance, in spite of the specific numerical learning difficulty of these children with MLD. For the children with RLD, number sense was a strong predictor for mathematics. Rapid naming too was a strong predictor for mathematics, fact retrieval and decoding. Their number sense scores were at the same level as TD-children’s performance. Rapid naming scores of children with RLD were weaker compared to TD-children. Finally, for the children with MRLD, number sense was a strong predictor for mathematics. There were no strong effects for fact retrieval and decoding. Symbolic number sense was the weakest in the MRLD-group compared to the other groups. Fact retrieval mediated the effect of rapid naming on mathematics in children with MLD and RLD, as well as the effect of number sense on mathematics in children with RLD.

### Follow-Up Analysis

To further examine whether rapid naming could be identified as compensatory mechanisms for mathematics, an exploratory Bayesian independent samples t-test was conducted in R using the BayesFactor-package (Morey et al., 2018). The previous analyses showed that number sense also predicted mathematics. Moreover, number sense has been indicated as an important marker of mathematics performance in the literature (Geary, 2011). Therefore, we first selected children whose number sense scores were ≤1 SD below the mean of the full sample. The full sample was used to avoid that selection of children with weak math scores might exclude those with strong compensatory mechanisms, who as a result do not fit with our selection criteria. Next, children were divided into two subgroups (−1 SD and +1 SD) based on their rapid naming scores: One group of children had weak number sense and weak rapid naming, and another group had weak number sense but strong rapid naming. Descriptive for both groups are displayed in Table 8.

**TABLE 8:** Background characteristics for the follow-up analyses.

	Weak number sense, strong rapid naming (*n* = 54)	Weak number sense, weak rapid naming (*n* = 61)
#MLD	9	2
#RLD	5	15
#MRLD	3	7
Working memory	0.65 (0.10)	0.62 (0.09)
Non-verbal reasoning	39.28 (7.75)	40.53 (5.81)

*Bayesian *t*-tests for working memory and non-verbal reasoning revealed that there were no initial group differences (*BF*s < 3). However, Bayesian Chi-square tests revealed that the number (#) of children with MLD, RLD, or MRLD per group was different (*BF*s > 3).*

The subgroups were compared on mathematics and on fact retrieval. Multivariate Bayesian analyses showed that the subgroups were different, *BF* = 3.98 (moderate support for H_1_; Jeffreys, 1961). A Bayesian *t*-test revealed that the subgroups differed on mathematics, *BF* = 4.77 (moderate support for H_1_; Jeffreys, 1961). Children with stronger rapid naming performed relatively better on mathematics (*M* = 9.20, *SD* = 6.33, *n* = 54) than children with weaker rapid naming (*M* = 8.10, *SD* = 5.80, *n* = 61). A Bayesian *t*-test for fact retrieval also suggested that the subgroups differed, *BF* = 2.11 (anecdotal support for H_1_; Jeffreys, 1961). Children with stronger rapid naming performed relatively better on fact retrieval (*M* = 116.13, *SD* = 30.73, *n* = 54) compared to children with weaker rapid naming (*M* = 106.31, *SD* = 33.51, *n* = 58).

## Discussion

Cognitive strengths were investigated in the present study as potential compensatory mechanism for primary school children’s cognitive weaknesses to partly overcome their learning difficulties in mathematics and/or reading. To elaborate, children with low mathematics performance seem to benefit from strong rapid naming skills to compensate for number sense and/or working memory weaknesses. A compensatory mechanism for a comorbid mathematics and reading learning difficulty was not identified in the present study.

Four groups were created using curriculum-based mathematics and reading scores in order to identify cognitive skills that could act as a compensatory mechanism for children with different ability levels. A Bayesian multigroup mediation SEM analyses showed that the model for typical developing (TD) children was consistent with the existing literature (see e.g., Friso-van den Bos et al., 2013; Araújo et al., 2015; Schneider et al., 2017 for meta-analyses). In short, mathematics was primarily predicted by number sense, working memory, non-verbal reasoning, and fact retrieval. Reading was primarily predicted by phonological awareness, rapid naming, and working memory. These findings are in line with our hypothesis. High achievers in any (combination) of those predictors, displayed higher performance on mathematics or reading as well.

It should be noted, however, that the direction of the effect of working memory on fact retrieval and reading was negative. In other words, weaker working memory skills were related to better fact retrieval and better decoding in TD-children. This somewhat unexpected result may be explained by means of a confounding variable. Working memory (or updating) can be viewed as an executive function (Miyake et al., 2000). The other executive functions of inhibition and shifting could be involved in fact retrieval and reading as well. Fact retrieval and reading both are timed measures, and a child will likely perform weaker on those tasks when he is, for example, distracted by task-irrelevant stimuli. When a child has to use much of his working memory resources on relatively simple tasks such as fact retrieval and reading, the efficacy of other executive functions might decrease, which may make him more prone to errors in those tasks.

Further it was expected that phonological processing (i.e., phonological awareness and/or rapid naming) could be identified as a cognitive compensation mechanism within children with mathematical difficulties. The results indeed showed that, within the MLD group, mathematics scores were better for children with relatively stronger rapid naming skills compared to peers with relatively weaker rapid naming skills. Note that the children with strength in rapid naming still performed worse on a mathematical task than children without any mathematical difficulties despite the compensatory effect from rapid naming.

Next, we investigated if our interpretation of the Bayesian SEM analyses holds: Does rapid naming also take on the role of a cognitive compensation in a regular primary school population? Performance on mathematics and reading was examined on a continuous dimensional scale for children with strong performance on this cognitive skill compared to children with weak performance on that same skill. The exploratory Bayesian *t*-tests in our full sample indeed confirmed our hypothesis that children with strong rapid naming (and a weak number sense as marker for mathematical difficulties) performed slightly better on mathematics and fact retrieval than children with weak rapid naming. Mean differences were small and standard deviations were large, thus cognitive compensation is not considered a mechanism that can resolve learning difficulties, and it may not apply to all children. Nevertheless, small gains in mathematics performance can be very meaningful for children with MLD. These results therefore point into the direction that rapid naming as a compensatory mechanism can reduce the severity of MLD.

For reading, a cognitive compensatory mechanism was not identified in the present study. It was hypothesized that strength in working memory might be a candidate for compensation, because previous research has shown that working memory is a less consistent predictor of reading compared to for example phonological awareness and rapid naming (Baddeley, 2003). However, this hypothesis was not supported. Working memory evidently is a prerequisite for reading (Savage et al., 2007), just like the other cognitive skills phonological awareness and rapid naming. Proficiency in certain (cognitive) skills may be essential for a child in order to be able to read. In contrast, MLD is a more heterogeneous learning disability (e.g., Price and Ansari, 2013), and for mathematics one may take alternative routes to acquire a minimum level of performance. Thus, there may be more possibilities for cognitive compensation in mathematics as opposed to reading. Nevertheless, strengths in other variables such as vocabulary (Haft et al., 2016), or affective variables such as motivation and self-esteem (Durlak et al., 2011) may be possible candidates for compensation of weaknesses related to reading. An alternative explanation for the lack of a compensatory mechanism for reading in the present study is the outcome measure that has been used. Reading was operationalized by (pseudo-)word decoding in the present study. However, a more complex task such as reading comprehension might appeal upon more cognitive skills, and may thus be more comparable with the complex problem solving task for mathematics. Indeed, previous research has shown that decoding is more associated with fact retrieval (De Smedt et al., 2010; Jordan et al., 2010), whereas reading comprehension is more associated with math problem solving (Pimperton and Nation, 2010; Björn et al., 2016). Thus, we cannot rule out the possibility that when a measure of reading comprehension had been included, a compensatory mechanism for reading could have been obtained.

Furthermore, there was a strong effect of number sense on mathematics within the children with reading difficulties. As not every child with a specific learning deficit develops a comorbid learning difficulty (i.e., children with reading difficulties performed better on mathematics than children with either specific or comorbid mathematical difficulties in the present study), we would like to suggest to the reader that the effect of number sense may be interpreted as a preventive mechanism for developing comorbid math difficulties. A strength in number sense might not compensate for cognitive weaknesses related to RLD *per se* (Moll et al., 2015a, b), but we speculate that it might prevent these children from the adverse effects of for example a phonological deficit. Such a deficit is of course related to reading difficulties and generally is also related to mathematical difficulties (Wilson et al., 2015). Due to a strong number sense, however, children may be able to avert this disadvantage by developing specific reading problems instead of a comorbid mathematics and reading learning difficulty.

With respect to children with a comorbid mathematical and reading learning difficulty (MRLD), results should be interpreted carefully. Although one of the advantages of Bayesian analyses is that it can be applied in small samples, any analyses with less than twenty children may be too small to detect an effect, especially for a complex model such as a multigroup mediation SEM (De Santis, 2007). We could therefore not confirm our hypothesis that compensation is not possible for children with MRLD. Nevertheless, there was little variance on the cognitive measures in the present study, as well as in previous research (Andersson, 2010), which demonstrates that children with MRLD are weak across the board. As these children show weaknesses on (almost) all cognitive skills related to their mathematics and reading performance, we carefully suggest that children with MRLD, who are known to have the most serious learning problem (Kaplan et al., 2006), are unable to compensate with a cognitive strength. This hypothesis should be tested in future research in a larger sample of children with MRLD.

The MRLD model also showed a relatively strong effect of number sense. Although this finding too should be interpreted with care, this is in line with the existing body of literature. It has previously been suggested that number sense can mainly be used to differentiate within children who are at the lower end of the continuum for mathematics (Geary et al., 2012). Variability in number sense skills cannot be used to distinct between children with strong math skills, because apparently all of them are able to solve these relatively simple numerical tasks. A child with very weak overall cognitive skills related to developing a mathematical and reading learning difficulty (i.e., the lower extreme of the continuum), might still benefit from slightly better number sense skills (compared to peers) when learning mathematics. Then again, the MRLD sample was quite small, thus future research should attempt to confirm this hypothesis by comparing children with MRLD with different levels of number sense in a larger sample size.

Previous research has demonstrated that the comorbidity between MLD and RLD likely occurs because of an overlap in the predictors of mathematics and reading. For instance, a child with weak phonological skills likely suffers from both mathematical and reading difficulties (Wilson et al., 2015). With respect to the cognitive compensation theory—as proposed in the present study—even children with comorbid learning difficulties might have a small cognitive strength. Low achievers could still perform slightly better on one of their cognitive skills compared to peers, despite limited variance in these cognitive skills, which can make their learning difficulty slightly less detrimental.

Finally, it is interesting to note that the Bayesian multigroup mediation SEM analyses showed the effect of several cognitive skills on mathematics to be mediated by fact retrieval. However, direct effects for most of the cognitive skills on mathematics (especially for working memory and non-verbal reasoning) were stronger than indirect effects via fact retrieval for TD-children. A potential explanation for this finding is that TD fourth-graders have already internalized the relatively simple arithmetic (fact retrieval) calculations (Mullis et al., 2016). Thus, when performing more complex math problem solving tasks, they might not need to rely much on their fact retrieval skills as these have already been automatized sufficiently. During these tasks, TD-children might instead invoke their cognitive resources such as working memory and non-verbal reasoning, because they are still learning new skills such as multiplying fractions. In line with a developmental framework, shifts may indeed occur over time within the relationship between various cognitive skills and mathematics (Van der Ven et al., 2013; Van de Weijer-Bergsma et al., 2015b).

While the mediation effect was positive for typical developing children, the effect was reversed for children with mathematical or reading difficulties in the multigroup model. Fact retrieval appeared to negatively mediate the effect of rapid naming on mathematics in children with MLD as well as in children with RLD. A similar negative mediation effect was obtained for number sense in children with RLD. This finding is surprising as correlations between fact retrieval and mathematics typically are strong and positive (see e.g., Träff, 2013), even in samples consisting of children with learning difficulties (see e.g., Träff and Samuelsson, 2013). Although this was not the main question of the present study, we would like to take the liberty to speculate about this unexpected negative mediation effect for fact retrieval. The correlation matrix provided no explanation as to why the effect was negative, thus this was probably a statistical artifact in the analyses due to the complexity of the model and given that this effect waned in the sensitivity analysis. It could also be speculated about a more conceptual explanation. The negative indirect effect could be interpreted as if stronger rapid naming (or number sense) is related to better fact retrieval skills, whereas better fact retrieval in turn appears to be related to worse math performance in children with specific learning difficulties. Number sense, rapid naming, and fact retrieval were all timed measures, thus a plausible explanation for this mediation can possibly be found in children’s processing speed. The finding that better fact retrieval is related to worse math performance might indicate that some children are able to perform quick numerical calculations because they have adequate processing speed skills (e.g., memorized knowledge, such as ‘3 ^∗^ 5 = __’), even though they do not grasp the meaning of problem solving tasks (e.g., understanding, such as ‘calculate the surface in millimeters of a 3 cm by 5 cm rectangle). Previous research has indeed shown that individual differences exist in children’s math performance. Some of them perform better on fact retrieval, whereas others are better in math problem solving (Huijsmans et al., under review). To add to that, it has been suggested that whereas typical math development involves progression from fact retrieval toward more procedural mathematics, some children with MLD tend to lag behind in this conceptual step (Thevenot, 2017; De Chambrier and Zesiger, 2018). This speculative explanation appears to be supported by the present study’s finding that rapid naming (and number sense) is positively related to fact retrieval for children with MLD. Thus, faster processing speed within children with specific learning difficulties (i.e., rapid naming for MLD, and rapid naming and number sense for RLD in the present study) might positively interfere with these children’s ability to quickly solve arithmetic facts, whereas it does not facilitate their procedural understanding of more complex problem solving tasks. However, conclusions regarding this finding should be investigated more thoroughly in future research considering the unexpected direction of the effects in comparison with previous literature.

To summarize, rapid naming is a likely candidate for cognitive compensation of number sense and possibly working memory weaknesses that are related to mathematical difficulties. Rapid naming is moderately related to mathematics (Berch and Mazzocco, 2007), which might explain why a strength in rapid naming takes on the role of a compensatory mechanism for mathematics for children with MLD as opposed to TD children. Regardless of children’s persistent difficulties with number sense and working memory, strength in rapid naming might enable children with MLD to partly overcome the possible negative effects of a cognitive deficit by taking an alternative route to learning mathematics compared to TD children. At this point we would like to take the liberty to speculate that a possible alternative route via rapid naming might call upon children’s general ability to retrieve facts from their long-term memory. These facts do not have to be numerical in nature *per se* (and they most likely are not entirely numerical because of those children’s weak number sense), but instead one might argue that they make more use of procedural facts. Fast and accurate retrieval (i.e., rapid naming) of procedural facts such as ‘when multiplying a rational number by ten, the decimal point is moved one place to the right’ might be initialized in some children with MLD, even when his conception of the magnitude of a series of numbers is imperfect. One possible interpretation thus is that children with weak number sense but strong rapid naming rely more on procedural strategies compared to children without a weak number sense. An alternative explanation for the strong association between rapid naming and mathematics for children with MLD is the role of language skills, such as grammar, vocabulary, decoding, and reading comprehension. Such language skills rely in part on rapid naming (Norton and Wolf, 2012), and have also been directly and indirectly related to mathematics (Björn et al., 2016). Direct associations with mathematics have been obtained for vocabulary (Kleemans et al., 2018) and reading comprehension (Björn et al., 2016), and can be explained by the fact that children apply their knowledge of math-words (such as ‘larger,’ ‘half,’ and ‘multiply’) when inferring the appropriate calculation from a word problem in the upper grades of primary school. Decoding has indirectly been associated with mathematics via fact retrieval, because children rely on retrieval of verbal codes from long-term memory during decoding and fact retrieval tasks, which is supported by rapid naming skills (Norton and Wolf, 2012; Koponen et al., 2017). As reading performance of the children with MLD is adequate, this might show that they have relatively strong cognitive skills related to reading. Proficiency in common precursors of mathematics–such as number sense–therefore does not seem to be a requirement for reaching a sufficient level of mathematics in primary school. Part of the delay in mathematics performance can be circumvented by a strength in related cognitive skills. Thus, children may to be able to partly reduce their mathematical learning disability.

This finding leads to a new direction in research on specific (mathematical) learning difficulties by suggesting that primary school children are to some extent able to compensate for their learning difficulties in the domains of mathematics. Likewise, similar mechanisms may exist in other academic domains such as reading and science. Equivalent to the theory of neural plasticity (Nelson, 1999), the conceptualization of cognitive compensation posits that a child who experiences a deficit in one process will rely more on another closely-related process to facilitate learning. The cognitive compensation theory leads to a different interpretation of the multiple deficit model of Pennington (2006) by including strengths beyond weaknesses. Strengths in this fashion were defined as relative to children with comparable characteristics (e.g., a group of children with math difficulties). Different conceptualizations, such as a relative strength within a child (e.g., average performance on a skill when performance on related skills is below average), are interesting to study in future research, because they might reflect individual variation even better. Nevertheless, this new multifactorial model re-conceptualizes our understanding of individual differences in learning: Each child with a specific learning difficulty has a unique profile of cognitive strengths and weaknesses with the goal to maximize their learning outcomes.

### Limitations and Suggestions for Future Research

At this point it should be mentioned that some of the measures used in the present study conveyed somewhat unexpected outcomes. First, fact retrieval skills were comparable across children with mathematical and/or reading difficulties. This may be a consequence of the speeded character of the test (i.e., processing speed; Berg, 2008), or underlying linguistic skills (Berch and Mazzocco, 2007). Secondly, children with mathematical difficulties surprisingly had the highest performance (i.e., quickest reaction times) on the non-symbolic number sense task. From the existing body of literature, however, it is evident that children with MLD at best perform equally to TD-children (Desoete and Grégoire, 2006). We hypothesize that the children with mathematical difficulties in the present study—possibly due to a lack of understanding—merely pressed one of the two buttons during the task, and therefore have faster reaction times compared to the other groups. Lastly, visuospatial working memory did not differ across groups, which contradicts the literature, wherein weaker visuospatial working memory usually is associated with lower math performance (Kroesbergen and Van Dijk, 2015). However, verbal working memory did differ across groups, which is in line with the notion that the effect of verbal working memory on mathematics performance increases as grade level progresses, while the effect of visuospatial working memory decreases (Van de Weijer-Bergsma et al., 2015b).

To add to the previous point, the BSEM model elicited some unexpected results as well. Some of the path coefficients for children with reading difficulties are inflated. This can likely be ascribed to the complexity of the model in relation to the sample size, and may be a statistical artifact (Lei and Wu, 2007). Even though the size or direction of some of the effects in the BSEM model were somewhat extreme, we are confident with our results given that these are mostly in line with previous studies. Nevertheless, future research might consider replicating these findings.

Third, it should be acknowledged that the present study consisted of a single measurement, and that we did not take a process measure of compensation into account. Causal inferences about the direction of the effects of cognitive strengths cannot be drawn from concurrent data (Hill and Stuart, 2015). Instead of using strong rapid naming skills to compensate for the detrimental consequences of weaknesses in cognitive predictors of mathematical difficulties (such as number sense), it might be the case that this strength arises from or co-occurs with reading proficiency in children with specific mathematical learning difficulties. Despite the reason for the strength of rapid naming in some children with mathematical difficulties, it seems plausible that children with weak mathematics performance might benefit from strong rapid naming skills. Future longitudinal research might shed light on the underlying mechanisms. For example, by using process measures during mathematics tasks (see e.g., Gidalevich and Kramarski, 2017), and by studying the patterns of correct and incorrect responses in mathematics tasks (see e.g., Koriakin et al., 2017). Children who use rapid naming to compensate for cognitive deficits will probably show patterns of correct responses on items that rely more on rapid naming (such as fact retrieval), whereas items that involve less rapid naming (but for example more number sense) may still be answered incorrectly.

A final point worthy of consideration is the question whether the compensatory effect is method-induced. To elaborate, variation within predictors may have shrunken substantially by selecting subsamples based on the outcome measures mathematics and reading. We considered the fact that this approach would be a restriction of range, but looking into the variance in minimum and maximum scores of the predictors lifted our concerns, because variance was substantial within groups as well as across groups. Group membership thus did not induce an artifact that could explain the compensatory effect in the present study. Nevertheless, it would be wise to replicate these findings in future research. Preferably first with a similar research design as proof of concept, and thereafter with different groups and variables related to learning, because compensation likely occurs in other domains as well.

## Conclusion

To conclude, the severity of mathematical learning difficulties might be reduced trough compensatory cognitive mechanisms, despite etiological factors (e.g., genes and environment) that confer risk for developing a specific learning disability. This leads to a more extensive view on learning difficulties (i.e., the cognitive compensation theory) compared to the multiple deficit model by Pennington (2006): Learning difficulties do not only result from several (cognitive) weaknesses, but seem to exist in combination with strengths in other skills. This is especially true for specific learning difficulties, but might apply to children with a comorbid learning difficulty as well. Mathematical performance is probably affected by cognitive strengths (i.e., rapid naming) in a reciprocal manner, which contributes to the individual’s ability to compensate to suboptimal circumstances. With the cognitive compensation theory, learning disability research is anticipated to shift from a restricted view of emphasizing an individual’s weaknesses toward the vision that each child has a unique profile of cognitive strengths and weaknesses, and that these strengths in one way or another may compensate for their weaknesses.

## Data Availability Statement

The datasets presented in this study can be found in online repositories. The names of the repository/repositories and accession number(s) can be found below: https://dx.doi.org/10.6084/m9.figshare.12122850.

## Ethics Statement

The studies involving human participants were reviewed and approved by Ethics Committee Social Science, Behavioural Science Institute, Radboud University. Written informed consent to participate in this study was provided by the participants’ legal guardian/next of kin.

## Author Contributions

MH, TK, and EK designed the research, analyzed and/or interpreted the data. MH performed the data-collection and wrote the manuscript. TK and EK gave feedback on draft versions. All the authors read and approved the final manuscript.

## Conflict of Interest

The authors declare that the research was conducted in the absence of any commercial or financial relationships that could be construed as a potential conflict of interest.

## Appendix

**TABLE A1:**

**Effect (X-Y)**	**Beta**	**N**	**SE**	**SD**	**Var**	**Precision**	**Source**	**Reason**	**Sample**
NS-FR	*0.38*	154	0.07	0.86	0.74	*1.36*	Kroesbergen and Van Dijk, 2015: Table 3, model 3 (p. 106)	Same tasks were used for NS and BA	Grade 2–5; TD + MLD
WM-FR	*0.36*	154	0.07	0.87	0.76	*1.31*	Kroesbergen and Van Dijk, 2015: Table 3, model 3 (p. 106)	Similar tasks were used for WM and BA	Grade 2–5; TD + MLD
PA-FR	*0.20*	167	0.07	0.96	0.93	*1.08*	Kleemans et al., 2018: Figure 1 (p. 410)	Comparable task was used for PA; same task for BA	Grade 5; TD
RAN-FR	*0.08*	103	0.10	1.00	1.00	*1.00*	Donker et al., 2016: data simulated based on paper	Same tasks were used for RAN and BA	Grade 1–5; TD + MLD + RLD + MRLD
NVR-FR	*0.11*	118	0.09	0.99	0.98	*1.02*	Filippetti and Richaud, 2017: Figure 4 (p. 877)	Comparable tasks were used for NVR and BA	Grade 2–6; TD
NS-Math	*0.34*	154	0.07	0.89	0.79	*1.27*	Kroesbergen and Van Dijk, 2015: Table 3, model 3 (p. 106)	Same task was used for NS; similar for AM	Grade 2–5; TD + MLD
WM- Math	*0.27*	154	0.08	0.93	0.87	*1.16*	Kroesbergen and Van Dijk, 2015: Table 3, model 3 (p. 106)	Similar tasks were used for WM and AM	Grade 2–5; TD + MLD
PA- Math	*0.33*	148	0.07	0.89	0.80	*1.25*	Slot et al., 2016 – Figure 1 (p. 7)	Similar tasks were used for PA and AM, but incl. BA	Grade 1–5; TD + MLD + RLD + MRLD
RAN- Math	*0.23*	103	0.09	0.95	0.91	*1.10*	Donker et al., 2016: data simulated based on paper	Same task was used for RAN; similar tasks for AM	Grade 1–5; TD + MLD + RLD + MRLD
NVR- Math	*1.20*	167	–0.03	–0.44	0.19	*5.13*	Kleemans et al., 2018: Figure 1 (p. 410)	Same tasks were used for NVR and AM	Grade 5; TD
FR- Math	*1.65*	167	–0.13	–1.73	2.98	*0.34*	Kleemans et al., 2018: Figure 1 (p. 410)	Same tasks were used for BA and AM	Grade 5; TD
WM-Dec	*–0.57*	91	0.07	0.68	0.46	*2.17*	Swanson et al., 2009: Tables 4, 5A,B (p. 268)	Meta-analysis	5-to-18-years-old; TD + RLD
PA-Dec	*0.74*	148	0.04	0.45	0.21	*4.85*	Slot et al., 2016: Figure 1 (p. 7)	Similar task was used for PA; same for Read	Grade 1–5; TD + MLD + RLD + MRLD
RAN-Dec	*0.44*	103	0.08	0.81	0.66	*1.52*	Donker et al., 2016: data simulated based on paper	Same tasks were used for RAN and Read	Grade 1–5; TD + MLD + RLD + MRLD
NVR-Dec	*0.11*	1335	0.03	0.99	0.98	*1.02*	Korpipää et al., 2017: Figure 2 (p. 136)	Comparable tasks were used for NVR and Read	Grade 1-7; TD

*Endogenous latent variables (Y’s): FR = Fact Retrieval; Math = Mathematics; Dec = (non-)word decoding. Exogenous latent variables (X’s): NS = Number Sense; WM = Working Memory; PA = Phonological Awareness; RAN = Rapid Automatized Naming; NVR = Nonverbal Reasoning. Values displayed in italics were used as priors. *Explanation reasons.* Same task, the source paper used the exact same task (or an older version) as the present study. Similar task, the task in the present study was based on/contained elements of the source task. Comparable task, the same construct was measured in both the source paper and in the present study.*
